# Langzeit‐Krebsüberleben (*Survivorship*) beim malignen Melanom

**DOI:** 10.1111/ddg.70486

**Published:** 2026-09-08

**Authors:** Markus Reitmajer, Susanne Dugas‐Breit, Chiara L. Blomen, Elisabeth Livingstone, Max Schlaak, Markus V. Heppt, Michael M. Sachse, Madlen Hörold, Friedegund Meier, Sebastian Haferkamp, Christian Apfelbacher, Georgia Schilling, Andrea von den Berg, Frank Meiss, Lisa Zimmer, Andrea Forschner

**Affiliations:** ^1^ Klinik für Dermatologie Universitätsklinikum Tübingen Tübingen Deutschland; ^2^ Abteilung für Medizinische Onkologie Nationales Zentrum für Tumorerkrankungen (NCT) Universitätsklinikum Heidelberg Heidelberg Deutschland; ^3^ Abteilung für Dermatologie und Venerologie Universitätsklinikum Hamburg‐Eppendorf (UKE) Hamburg Deutschland Fleur‐Hiege‐Zentrum für Hautkrebsforschung Universitätsklinikum Hamburg‐Eppendorf (UKE) Hamburg Deutschland; ^4^ Klinik für Dermatologie Universitätsklinikum Essen Essen Deutschland; ^5^ Klinik für Dermatologie Venerologie und Allergologie Charité‐Universitätsmedizin Berlin angegliedert an die Freie Universität Berlin und die Humboldt‐Universität zu Berlin Berlin Deutschland; ^6^ Klinik für Dermatologie Uniklinikum Erlangen Friedrich‐Alexander‐Universität Erlangen‐Nürnberg (FAU) Erlangen Deutschland; ^7^ Comprehensive Cancer Center Erlangen‐Europäische Metropolregion Nürnberg (CCC ER‐EMN) Erlangen Deutschland Bayerisches Zentrum für Krebsforschung (BZKF) Uniklinikum Erlangen Friedrich‐Alexander‐Universität Erlangen‐Nürnberg (FAU) Erlangen Deutschland; ^8^ Dermpath München Labor für Dermatopathologie Oralpathologie und Molekulare Pathologie München Deutschland; ^9^ Klinik für Dermatologie Allergologie und Phlebologie Bremerhaven Reinkenheide Bremerhaven Deutschland; ^10^ Institut für Sozialmedizin und Gesundheitssystemforschung Medizinische Fakultät Otto‐von‐Guericke‐Universität Magdeburg Magdeburg Deutschland; ^11^ Hautkrebszentrum am Universitätsklinikum Dresden und Nationales Zentrum für Tumorerkrankungen Dresden Deutschland; ^12^ Klinik für Dermatologie Medizinische Fakultät und Universitätsklinikum Carl Gustav Carus Technische Universität Dresden Dresden Deutschland; ^13^ Klinik für Dermatologie Universitätsklinikum Regensburg Regensburg Deutschland; ^14^ Klinik für Dermatologie Universitätsklinikum und Medizinische Fakultät Martin‐Luther‐Universität Halle‐Wittenberg Halle (Saale) Deutschland; ^15^ Abteilung für onkologische Rehabilitation Asklepios Nordseeklinik Westerland, Deutschland; ^16^ Abteilung für onkologische Rehabilitation Asklepios Klinik Triberg Triberg Deutschland; ^17^ Abteilung für Dermatologie Medizinisches Zentrum – Universität Freiburg Medizinische Fakultät Universität Freiburg Freiburg Deutschland

**Keywords:** gesundheitsbezogene Lebensqualität (HRQoL), immunbedingte Nebenwirkungen (irAEs), Immun‐Checkpoint‐Inhibitor (ICI), Lebensqualität (QoL), Melanom, Nachsorge, Überleben, Überwachung, follow‐up, health‐related quality of life HRQoL, immune‐related adverse events (irAEs), immune checkpoint inhibitor (ICI), melanoma, quality of life (QoL), survivorship, surveillance

## Abstract

Für das metastasierte Melanom liegen inzwischen robuste Daten zum Langzeitüberleben vor; so beträgt unter kombinierter Therapie mit Immuncheckpointinhibitoren (ICI) im metastasierten Stadium die melanomspezifische 10‐Jahres‐Überlebensrate über 50 %. Auch für Patienten mit Hirnmetastasen ist dauerhafte Krankheitskontrolle inzwischen bei einem substantiellen Anteil der Patienten erreichbar.

Mit zunehmendem Langzeitüberleben, insbesondere nach zuvor erfolgter Therapie mit ICI, rücken chronische und spät auftretende therapieassoziierte Toxizitäten vermehrt in den Fokus, insbesondere chronische irAEs, die auch nach Beendigung einer Therapie mit ICI persistieren oder auch erst beginnen können. Langzeittoxizitäten können die Lebensqualität nachhaltig beeinträchtigen und erfordern zum Beispiel bei endokrinologischen Toxizitäten mitunter eine lebenslange Substitution. Neben den unmittelbar physischen Aspekten, sind häufig psychosoziale und finanzielle Belastungen, Schwierigkeiten beim beruflichen Wiedereinstieg, sowie Aspekte der Familienplanung und Fertilität zentrale Survivorship‐Themen. Die systematische Erfassung sämtlicher psychosozialer und physischer Belasungen, sowie der Lebensqualität, insbesondere als patientenberichtetes Outcome unterstützt Therapieentscheidungen und Nachsorgeplanung und sollte Teil des multidisziplinären *Survivorship‐Care*‐Konzeptes sein.

## DEFINITION UND TERMINOLOGIE DES LANGZEITÜBERLEBENS (*SURVIVORSHIP*)

Die Anzahl der Menschen, die eine Krebserkrankung überleben, steigt kontinuierlich.^1^ Die Ursachen hierfür sind vielfältig und multifaktoriell. Neben der steigenden Lebenserwartung und dem demographischen Wandel führen auch verbesserte Früherkennungsprogramme, optimierte Diagnostik und innovative Therapiemöglichkeiten zu signifikant verbessertem Überleben nach einer Krebsdiagnose. Nach aktuellen Schätzungen leben in den USA nahezu 1,5 Millionen Menschen mit und nach einem Melanom,^1^ während in Deutschland mehr als 300 000 Menschen mit und nach dieser Erkrankung leben.^2^

Zwei Drittel dieser Melanom‐Überlebenden in Deutschland sind Langzeitüberlebende, bei denen die Erstdiagnose mindestens fünf Jahre zurückliegt. Fast die Hälfte dieser Menschen befindet sich im erwerbsfähigen Alter beziehungsweise sind jünger als 65 Jahre.^2^ Eine vergleichbare Situation zeigt sich in den USA, wo mehr als 70 % der Melanom‐ Überlebenden zu den Langzeitüberlebenden zählen und 40 % im arbeitsfähigen Alter, 12 % jünger als 50 Jahre sind.^1^

### *Cancer Survivor* und Langzeitüberleben

**ABBILDUNG 1:**
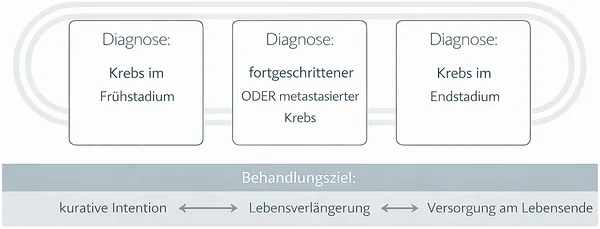
Versorgungsziele im Verlauf einer Krebserkrankung. Die Abbildung zeigt den zeitlichen Verlauf einer Krebserkrankung von der Frühphase über das fortgeschrittene oder metastasierte Stadium bis hin zur Endphase der Erkrankung. Dargestellt sind die jeweils dominierenden Versorgungsziele, die sich im Krankheitsverlauf von kurativer Intention über lebensverlängernde Therapieansätze bis hin zur palliativen Versorgung verschieben. Der Übergang zwischen den Phasen ist dabei fließend und kann individuell variieren. Die Abbildung wurde mithilfe der Software abacus.ai (Abacus.AI, San Francisco, CA, USA) erstellt.

Ein *Cancer Survivor* ist grundsätzlich jeder Mensch vom Zeitpunkt der Krebsdiagnose bis zu seinem Lebensende, unabhängig von Krankheitsstadium, Prognose oder Krankheitsverlauf.^3^ *Cancer Survivor* sind somit eine heterogene Gruppe von Menschen, die sich in unterschiedlichen Phasen der Krebserkrankung befinden und daher auch unterschiedliche Bedürfnisse sowie individuellen Unterstützungsbedarf haben (Abbildung 1).^4^ Im Kontext des Melanoms bedeutet dies, dass zu den Melanom‐ Überlebenden sowohl Menschen mit Erstdiagnose eines Melanoms nach vollständiger Tumorentfernung als auch Menschen mit fortgeschrittener Erkrankung gehören, die beispielsweise eine systemische Therapie erhalten. Von Langzeitüberlebenden spricht man in der Regel, wenn die Erstdiagnose der Krebserkrankung mindestens 5 Jahre zurückliegt.^5^

### Langzeitüberlebensdaten beim Melanom

Beim Melanom liegen mittlerweile fundierte 10‐Jahres‐Überlebensdaten unter Immuncheckpoint‐Inhibitor (ICI) Therapie aus zwei wegweisenden Phase‐3‐Studien vor.^6^, ^7^ In der Checkmate 067‐Studie zeigten therapienaive Patienten mit metastasiertem Melanom unter kombinierter Therapie mit Ipilimumab und Nivolumab ein 10‐Jahres‐Gesamtüberleben von 43 % und unter Nivolumab‐Monotherapie von 37 %.^6^ Das 10‐Jahres‐Melanom‐spezifische Überleben lag bei 52 % unter kombinierter ICI und bei 44 % unter Nivolumab‐Monotherapie. Bemerkenswert ist, dass Patienten, die drei Jahre nach Beginn der Therapie mit Ipilimumab und Nivolumab oder Nivolumab‐Monotherapie keinen Progress aufwiesen, ein Melanom‐spezifisches Überleben von ≥ 96 % zeigten. Als weiterer wichtiger Prädiktor für das Langzeitüberleben erwies sich die Tiefe des Ansprechens. Patienten mit einer ≥ 80 %igen Tumorreduktion zeigten ein 10‐Jahres‐Melanom‐spezifisches Überleben von ≥ 87 %.^6^

Vergleichbare Ergebnisse zeigten sich in der Keynote 006‐Studie.^7^ Patienten, die mit dem Anti‐PD1‐Antikörper Pembrolizumab behandelt wurden, zeigten ein 10‐Jahres‐Gesamtüberleben von 34 % und ein melanomspezifisches 10‐Jahres‐Überleben von 45 %. Analog zur Checkmate 067‐Studie hatten Patienten mit eine Komplettremission (CR) das günstigste 8‐Jahres‐Melanom‐spezifische Überleben mit 88 % im Vergleich zu 68 % bei partiellem Tumoransprechen (PR) und 36 % bei stabiler Erkrankung (SD).^7^ Ein weiterer wichtiger Aspekt der Keynote 006‐Studie ist die Erkenntnis, dass Patienten die ICI nicht dauerhaft fortführen müssen, um ein Langzeitüberleben zu erreichen. So waren 65 % der Patienten, die Pembrolizumab nach zwei Jahren aufgrund von CR/PR/SD beendet hatten, nach acht Jahren noch ohne Progress und mehr als 80 % am Leben.^7^

In der Checkmate 067‐Studie zeigten therapienaive Patienten mit metastasiertem Melanom unter kombinierter Therapie mit Ipilimumab und Nivolumab ein 10‐Jahres‐Gesamtüberleben von 43 %, das melanomspezifische 10‐Jahres‐Überleben lag bei 52 %. Bei einer so langen Nachbeobachtungszeit hat das melanomspezifische Überleben einen besonders hohen Stellenwert.^6^

Auch mit einer PD‐1 Antikörper Therapie als Monotherapie sind langandauernde Remissionen zu erreichen.^6^ So lag das melanomspezifische 10‐Jahres‐Überleben bei 44 % beziehungsweise 45 % mit einer Nivolumab‐ beziehungsweise Pembrolizumab Monotherapie.^6^, ^7^

### Langzeitdaten bei Hirnmetastasen: Die ABC‐Studie

Eine besondere klinische Herausforderung stellen Hirnmetastasen beim Melanom dar, die bei 30‐40 % der Patienten auftreten. Auch für diese besondere Patientengruppe liegen nun Langzeitdaten vor. In der australischen multizentrischen randomisierten Phase‐2‐Studie „Anti‐PD‐1 Brain Collaboration“ (ABC) zeigten ICI‐naive Patienten mit asymptomatischen Hirnmetastasen ein 7‐Jahres‐Gesamtüberleben unter kombinierter ICI mit Ipilimumab/Nivolumab von 48 % im Vergleich zu 26 % unter Nivolumab‐Monotherapie.^8^ Die intrakranielle Ansprechrate betrug 51 % für die Kombinationstherapie gegenüber 20 % für die Nivolumab‐Monotherapie.^8^ Die Daten unterstreichen eindrucksvoll, dass bei einem substanziellen Anteil der Patienten mit Hirnmetastasen eine langfristige Krankheitskontrolle durch Ipilimumab/Nivolumab erreicht werden kann.

## CHRONISCHE UND SPÄTE TOXIZITÄTEN UNTER IMMUN‐ UND ZIELGERICHTETER THERAPIE BEIM MELANOM

### Chronische und späte Toxizitäten unter Immuntherapie

Mit der zunehmenden Zahl langzeitüberlebender Melanompatienten werden persistierende und spät auftretende therapieassoziierte Toxizitäten zunehmend relevant. Während akute immunvermittelte Nebenwirkungen (*immune‐related adverse events*, irAEs) unter ICI gut charakterisiert sind, wurde ihre Chronifizierung lange unterschätzt. Aktuelle prospektive und retrospektive Analysen zeigen jedoch, dass ein relevanter Anteil der Behandelten unter anhaltenden funktionellen Einschränkungen leidet, die die Lebensqualität (QoL) dauerhaft beeinträchtigen können. Für die klinische Praxis und die Vergleichbarkeit von Studien sowie eine systematische Erfassung von Spättoxizitäten ist eine einheitliche Terminologie essenziell. Die *Society for Immunotherapy of Cancer* (SITC) definierte „chronische irAEs“ in einem internationalen Konsensuspapier als Nebenwirkungen, die drei Monate nach Absetzen der ICI‐Therapie persistieren. Zusätzlich wird unterschieden in „chronisch‐aktive“ und „chronisch‐inaktive“ irAEs in Abhängigkeit davon, ob eine anhaltende Entzündung vorliegt und/oder eine immunsuppressive Therapie benötigt wird.^9^ Als „verzögerte“ oder „*late‐onset* irAE“ gelten Nebenwirkungen, die innerhalb von sechs Monaten nach Therapieende auftreten. Gerade bei den Nebenwirkungen, die nach Beendigung einer Therapie auftreten, ist die kausale Rückführung auf die ICI schwierig, insbesondere dann, wenn die Patienten Folgetherapien erhalten haben.

Aktuelle prospektive und retrospektive Analysen zeigen jedoch, dass ein relevanter Anteil der Behandelten unter anhaltenden funktionellen Einschränkungen leidet, die die Lebensqualität (QoL) dauerhaft beeinträchtigen können.

Die bislang detailliertesten Daten zur Chronifizierung von irAEs beim Melanom stammen aus einer Kohorte adjuvant behandelter Hochrisikopatienten unter Anti‐PD‐1‐Therapie. In dieser retrospektiven Untersuchung zeigte sich, dass von 387 adjuvant mit Anti‐PD‐1‐Therapie behandelten Patienten 43 % mindestens ein chronisches irAE entwickelt hatten.^10^ In der verlängerten Nachbeobachtung dieser Kohorte ^11^ persistierten bei rund 29 % der Betroffenen die Symptome länger als 1,5 Jahre nach Therapieende, ca. 45 % der chronischen irAEs waren weiterhin symptomatisch. Besonders häufig zeigten sich adrenale Insuffizienz, Hypothyreose und Thyreoiditis, Neuropathie und Nephritis chronifiziert. Fatigue wurde ebenfalls häufig von den Patienten angegeben, die Autoren hatten sich jedoch entschlossen, Fatigue nicht mit in die Statistik einzubeziehen, da die kausale Zuweisung zur ICI schwierig ist. In einer aktuellen deutschen Studie unter Langzeit‐Melanomüberlebern (≥ 5 Jahre nach Erstdiagnose Stadium IV), gaben mehr als 40 % der Patienten chronische Nebenwirkungen an.^12^

**ABBILDUNG 2:**
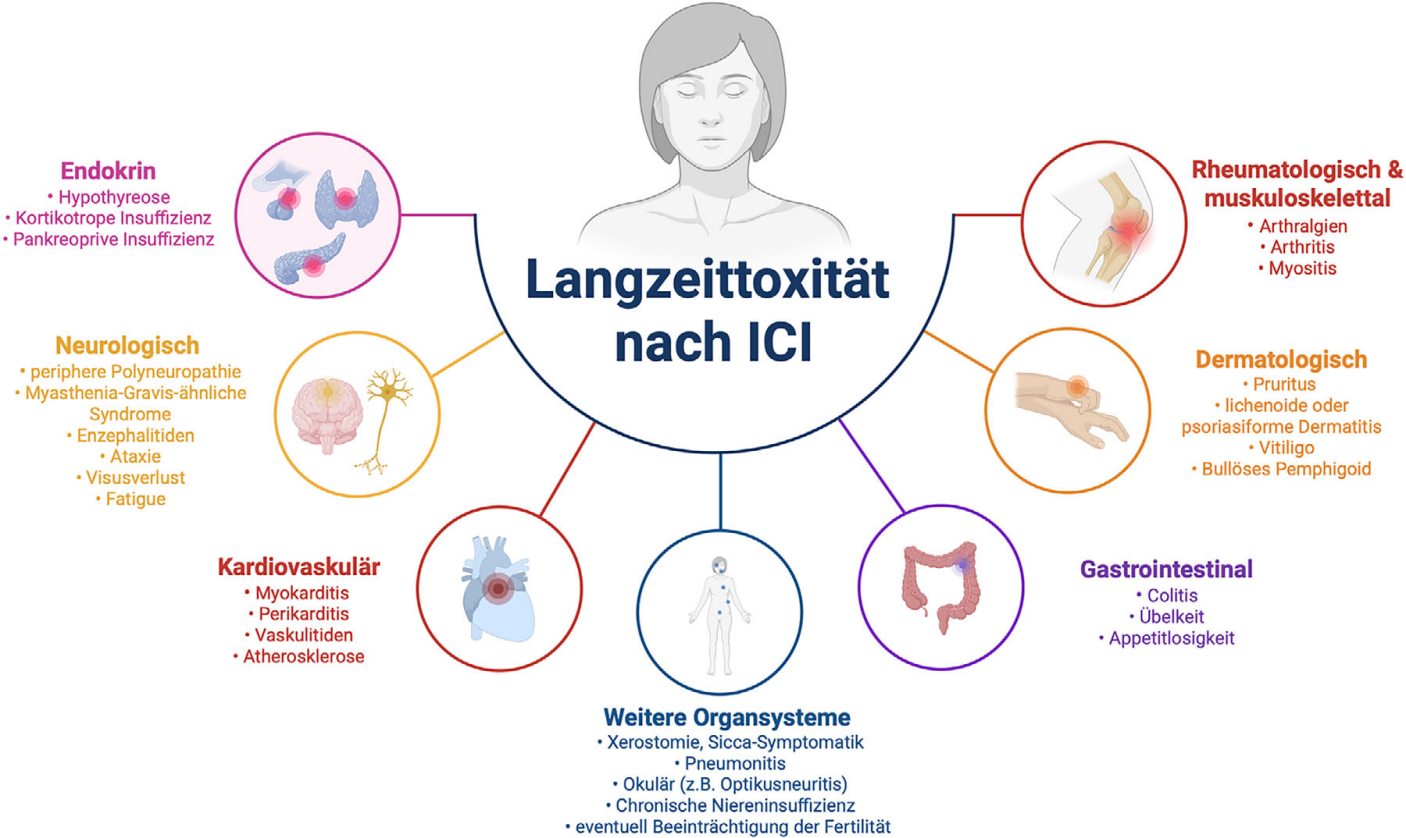
Langzeittoxizitäten nach Immuncheckpoint‐Inhibitor (ICI) Therapie. Die Abbildung veranschaulicht die häufigsten Langzeittoxizitäten nach Behandlung mit Immuncheckpoint Inhibitoren (ICI). Typische klinische Manifestationen werden dargestellt, wobei nach betroffenen Organsystemen differenziert wird. Die Abbildung wurde mithilfe der Software BioRender (Toronto, Kanada) erstellt.

Systematische Übersichten bestätigen, dass chronische irAEs über Entitäten hinweg klinisch bedeutsam häufig sind und in vielen Studien eher unterschätzt wurden.^13^, ^14^ Die Mechanismen chronischer irAEs sind bislang unvollständig verstanden. Diskutiert werden irreversible Organschäden in der akuten Phase (insbesondere bei endokrinologischen irAE), persistierende Entzündungen (irArthritis) und grundsätzliche Mechanismen, die zur Entstehung von irAE führen (Kreuzreaktivität zwischen Tumor‐ und Eigenantigenen, Umwelteinflüsse, das Mikrobiom, smoldering (schwelende) Autoimmunität, gewebsassoziierte Immunzellen und genetische Faktoren).^14^ In Tabelle 1 und Abbildung 2 sind die für die Praxis wichtigsten organbezogenen chronischen/Spät‐Toxizitäten, typische Persistenzmuster, Monitoring und Management zusammengefasst.^10^, ^11^, ^13^, ^14^, ^15^, ^16^, ^17^, ^18^

**TABELLE 1:** Organbezogene chronische irAEs – Häufigkeit – Persistenz – Management. Die Häufigkeitsangaben variieren je nach Kollektiv und Definition chronischer irAEs.

Organsystem	Typische chronische/Spättoxizitäten	Häufigkeit/Beispiele (melanomspezifisch, sofern verfügbar)	Persistenzmuster	Monitoring in der Nachsorge	Management (Survivorship‐orientiert)	Zentrale Referenzen
**Endokrin (ICI)**	Hypothyreose/Thyreoiditis, Hypophysitis, Nebenniereninsuffizienz, (seltener) Diabetes	Hypothyreos/Thyreoiditis chronisch 14 % (davon 85,7 % > 1,5 Jahre persistierend); Hypophysitis/Nebenniereninsuffizienz in Kohorte vollständig persistierend	meist **irreversibel** (chronisch‐inaktiv)	TSH/fT4, Cortisol/ACTH, Elektrolyte, HbA1c/Glukose; patientenberichtete Symptome	Dauerhafte Substitution; Notfallausweis/Stressdosis‐Schulung; koordinierte Endokrinologie	^11^, ^14^, ^18^
**Rheumatologisch (ICI)**	Chronische Arthritis/Arthralgien, Myositis‐Overlap, PMR‐ähnlich	Arthritis/Arthralgien chronisch 5,7 %; 41 % > 1,5 Jahre persistierend	häufig **rezidivierend/ steroidabhängig**	Funktion/Schmerz, Entzündungsmarker, ggf. Bildgebung	Steroid‐sparend (DMARDs/Biologika), Physio/Schmerzmedizin	^11^, ^14^, ^18^
**Gastrointestinal (ICI)**	Chronische Kolitis/Enteritis, mikroskopische Kolitis, Malabsorption	In GI‐irAE‐Kohorte 8 % mit Entzündung ≥ 6 Monate nach ICI; in Adjuvanz‐Kohorte Kolitis selten chronisch	Monate bis Jahre; teils **steroidrefraktär**	Stuhl‐Anamnese, Calprotectin, Endoskopie bei Persistenz/Rezidiv	Eskalation: Steroide → Vedolizumab/Infliximab u. a.; Ernährung/Supportiv; selten Chirurgie	^11^, ^15^
**Neurologisch (ICI)**	Neuropathien, Myositis/MG‐Overlap, Enzephalitis/Myelitis, autonome Störungen	selten (< 5 %); in n‐irAE‐Kohorte: 57 % chronisch (nicht‐fulminant), ∼52 % davon chronisch‐aktiv	hohe Rate **Residualdefizite**; Teil chronisch‐aktiv	Neurologische Funktionsscores, Reha‐Bedarf; Screening Myokarditis bei Myositis/MG	Frühe Hochdosis‐Steroide, IVIG/PLEX, ggf. Rituximab; Rehabilitation; interdisziplinär	^16^
**Dermatologisch (ICI)**	Chronischer Pruritus/Dermatitis, lichenoid/psoriasiform, BP‐ähnlich; Vitiligo	häufig (10–20 %); Dermatitis/Pruritus chronisch 6,6 % (22,1 % > 1,5 Jahre persistierend); chronische kutane irAE‐Dauer median ∼629 d, Persistenz 54 % nach 18 Monaten	häufig persistierend; BP‐ähnlich besonders langwierig	Dermatologische Nachuntersuchungen, PRO‐Erfassung Pruritus/Schlaf	Topisch/systemisch; steroid‐sparend (z. B. Biologika) bei ausgewählten Mustern	^11^, ^17^
**Kardiovaskulär (ICI)**	Myokarditis (inkl. subklinisch/„smoldering“), Perikarditis, Vaskulitis, Arrhythmien; potenziell atherosklerotische Ereignisse/Plaque‐Progression	Chronische kardiale irAEs insgesamt selten (in Reviews ca. 2 % der nicht‐endokrinen chronischen irAEs); in adjuvanter Anti‐PD‐1‐Kohorte chronische Myokarditis 0,3 % (persistierend)	Langzeitverlauf nach überstandener Myokarditis/Perikarditis nicht gut definiert (Risiko persistierender inflammatorischer Kardiomyopathie bzw. später LV‐Dysfunktion unklar); mögliches unterschätztes Risiko für atherosklerotische Ereignisse	Niedrigschwellige Kardioonkologie; bei Symptomen/auffälligen Biomarkern: EKG, Troponin/BNP, Echo, ggf. Kardio‐MRT; zusätzlich konsequente CV‐Risikoprofilanalyse (RR, Lipide, Diabetes, Rauchen), ggf. Verlauf je nach Risikokonstellation	chronische Residuen: HF‐Therapie/Arrhythmie‐Management; bei möglicher Atherosklerose: intensives Risikofaktormanagement (Statin/Antihypertensiva/Diabeteskontrolle), Bewegung/Reha	^10^, ^13^, ^18^
**Speicheldrüsen/oral (ICI)**	Xerostomie/Sicca (Sjögren‐ähnlich)	Xerostomie chronisch 2,3 %; 88,9 % persistierend	meist langanhaltend, häufig chronisch‐inaktiv	Mund‐/Augensymptome, Infekt‐/Karies‐Anamnese; zahnärztliche Kontrollen	Speichelersatz/‐stimulation, Fluoridierung; ggf. Sialogoga (Pilocarpin/Cevimelin); Zahnmedizin/ggf. Rheumatologie	^10^
**Pulmonal (ICI)**	Pneumonitis, persistierende radiologische Veränderungen	Pneumonitis chronisch 2,5 % (44,4 % > 1,5 Jahre persistierend)	variabel; Rezidive möglich	Klinik, low‐dose CT, Lungenfunktionsdiagnostik, bronchoalveläre Lavage	Steroide; differenzialdiagnostisch Infektionen/Tumorprogress	^10^, ^11^
**Renal (ICI)**	Nephritis, CKD	Nephritis/nephrotisches Syndrom chronisch 1,3 % (50 % > 1,5 Jahre persistierend)	teils persistierend	Kreatinin/eGFR, Urinstatus, Mikroproteinanalyse	Steroide/Immunsuppression; Nephrologie	^10^, ^11^
**Okulär (ICI)**	Uveitis, Optikusneuritis, retinale Vaskulitis	okuläre chronische irAEs 1,3 % (57,1 % > 1,5 Jahre persistierend)	variabel	ophthalmologisch symptombezogen/bei Vorgeschichte	Topisch/systemisch; Ophthalmologie	^16^, ^18^

### Spättoxizität unter zielgerichteter Therapie

Auch BRAF‐ und MEK‐Inhibitoren können zu chronischen Nebenwirkungen führen, wenngleich diese deutlich seltener und meist nicht irreversibel sind. In einer großen multizentrischen retrospektiven Adjuvanz‐Kohorte (n = 598; Stadium III, BRAF V600‐mutiert) wurden persistierende Nebenwirkungen (> 3 Monate nach Therapieende) unter Dabrafenib/Trametinib bei 3 % angeben, unter Anti‐PD‐1 dagegen bei 26 %. Arthralgien sowie kardiovaskuläre Effekte (zum Beispiel. linksventrikuläre Dysfunktion unter MEK‐Inhibitoren) können die QoL langfristig beeinträchtigen, bilden sich nach dem Absetzen der zielgerichteten Therapie (TT) jedoch meist zurück. Ebenso sind retinale Ödeme (MEK‐assoziierte Retinopathien) in der Regel transient, anhaltende okuläre Nebenwirkungen wie ein Retinalvenenverschluss treten sehr selten auf. *Patient‐reported‐outcome*‐Daten aus der Adjuvanz zeigen, dass auch unter Dabrafenib/Trametinib chronische Fatigue auftreten kann.^19^

### Finanzielle Toxizität, berufliche Reintegration und Leistungsfähigkeit

Chronische und späte Toxizitäten führen häufig zu dauerhaften Folgekosten (Hormonsubstitution, wiederholte Facharztkontakte, Biologika/Immunsuppressiva, Rehabilitation). Zusätzlich kann ein durch die Toxizitäten bedingter Arbeitsausfall die finanzielle Belastung weiter verstärken. Gerade chronisch‐aktive irAEs mit Langzeit‐Immunsuppression oder Biologika‐Bedarf sind ressourcenintensiv.

Für die berufliche Reintegration sind weniger seltene Organschäden als vielmehr persistierende Symptome (Fatigue, Schmerz, GI‐Beschwerden, dermatologischer Pruritus) limitierend. In einer australischen Studie gaben 50 % der Patienten an, ein Jahr nach der Beendigung einer adjuvanten ICI‐/TT‐Therapie weiterhin eine reduzierte Arbeitszeit und ein reduziertes Einkommen zu haben; ein Drittel berichtete finanzielle Toxizität.^20^ Ähnliche Fragestellungen gewinnen zunehmend auch im deutschsprachigen Raum an Bedeutung. So betont die deutsche S3 Melanom‐Leitlinie explizit die Relevanz beruflicher Wiedereingliederung und sozialmedizinischer Rehabilitation im Langzeitverlauf.^21^ Vergleichbare Daten aus Deutschland sind derzeit nicht publiziert (Stand 05/2026).

## FERTILITÄT, FAMILIENPLANUNG UND SCHWANGERSCHAFT

Das maligne Melanom zählt zu den häufigsten Krebserkrankungen bei jungen Erwachsenen im gebärfähigen Alter.^22^, ^23^ Angesichts der inzwischen erreichten Langzeitüberlebensraten rücken die Familienplanung und der damit verbundene Erhalt der Fertilität zunehmend in den Fokus der onkologischen Versorgung.

### Auswirkungen systemischer Therapien beim Melanom auf die Fertilität

Für einen Großteil der Betroffenen stellt der Verlust der Fertilität eine große Sorge vor dem Beginn einer Krebstherapie dar und kann so belastend wie die Krebsdiagnose selber sein.^24^ Robuste Daten zur Fertilität nach ICI oder BRAF/MEK‐Therapie sind limitiert, die tatsächlichen Auswirkungen auf die Fertilität noch unklar.^23^, ^26^ Eine Beeinträchtigung der Fertilität kann nach der vorliegenden Fachinformation nicht ausgeschlossen werden.^25^ Eine verminderte männliche Fertilität unter und nach TT kann möglich sein. Zudem ist zu bedenken, dass aufgrund potenzieller fetaler Risiken und begrenzter Evidenz eine konsequente Kontrazeption während einer systemischen Therapie und empfohlen wird; dies gilt insbesondere für TT.

Für ICI liegen ebenfalls wenig Daten zur potentiellen Gonadentoxizität vor. In einer retrospektiven Analyse in einer Nebenwirkungsdatenbank der US Food and Drug Administration (FDA) wurden 133 512 Patienten mit ICI‐Therapie untersucht, bei 0,43 % entwickelten sich Nebenwirkungen am Reproduktionssystem. Die Nebenwirkungsrate war bei Frauen höher als bei Männern (0,57 % vs. 0,39 %, p < 0,001). Für Frauen wurden vaginale Blutungen und 34 Fälle von Fistelbildungen im Genitaltrakt beschrieben. Bei Männern wurde am häufigsten über Veränderungen der Spermiogenese berichtet, vor allen nach einer dualen ICI‐Therapie mit PD‐1 plus CTLA‐4 Antikörper, aufgrund der geringen Patientenzahl (n = 6) ist die Aussagefähigkeit dieser Daten jedoch begrenzt.^26^

Die Fruchtbarkeit kann auch indirekt beeinträchtigt werden. Insbesondere endokrine irAEs wie Hypophysitis, Thyreoiditis oder eine primäre Gonadeninsuffizienz könnten zu hormonellen Störungen führen, die sich negativ auf die Reproduktionsfähigkeit auswirken. Diese hormonellen Veränderungen sind im Regelfall durch Substitution im Rahmen der endokrinologischen Betreuung behandelbar.

### Fertilitätsberatung vor Beginn der medikamentösen Therapie

Internationale Leitlinien und Empfehlungen von Experten betonen die Bedeutung einer frühzeitigen, strukturierten Fertilitätsberatung bei allen Patienten im reproduktiven Alter vor Beginn einer systemischen onkologischen Therapie.^27^, ^28^ Diese Beratung sollte individuell erfolgen und Aspekte wie Kinderwunsch, Prognose, therapiebedingte Risiken für die Fertilität, notwendige Kontrazeptionsmaßnahmen sowie verfügbare Optionen zum Fertilitätserhalt umfassen. Eine zeitnahe Überweisung an spezialisierte reproduktionsmedizinische Zentren wird empfohlen. Zusätzlich sollte psychosoziale Unterstützung angeboten und die Beratung dokumentiert werden.

### Möglichkeiten des Fertilitätserhalts

Nach der AWMF‐S2k‐Leitlinie zum Fertilitätserhalt bei onkologischen Erkrankungen werden bei Frauen die Kryokonservierung fertilisierter oder unfertilisierter Oozyten sowie von Ovarialgewebe empfohlen; in ausgewählten Fällen kann zudem der Einsatz von GnRH‐Agonisten erwogen werden.^29^ Die Oozytenkryokonservierung erfordert eine etwa zweiwöchige ovarielle Stimulation mit anschließender Eizellentnahme, während die Entnahme von Ovarialgewebe kurzfristig erfolgen und zu einem späteren Zeitpunkt retransplantiert werden kann. Bei Männern wird primär die Kryokonservierung von Ejakulat empfohlen; in Einzelfällen kommt auch die Kryokonservierung von Hodengewebe infrage.^22^, ^26^, ^29^

### Schwangerschaftsmanagement

Für BRAF‐ und MEK‐Inhibitoren liegen bislang nur wenige klinische Daten vor, jedoch weisen tierexperimentelle Studien auf embryo‐ und fetotoxische sowie teratogene Effekte hin. Der Einfluss von TT‐und ICI‐Therapien im Rahmen der Schwangerschaft weitgehend unklar. In einer retrospektiven Untersuchung wurde über 49 Frauen unter ICI und potentiellen Auswirkungen auf die Reproduktionsorgane berichtet. In 45 % der Fälle hatten die Patienten eine Therapie mit Nivolumab als Monotherapie erhalten. In dieser Kohorte kam es zu neun Konzeptionen, zwei unter laufender Therapie mit einer Fehlgeburt und sieben Schwangerschaften nach Ende der Therapie, hierbei traten zwei spontane Aborte und ein elektiver Abort auf. ^30^

Während und je nach Zulassung 4–5 Monate nach einer systemischen Melanomtherapie ist eine zuverlässige Kontrazeption erforderlich, da potenzielle Risiken für den Embryo/Fötus bestehen können. Ebenfalls kann die Effektivität der oralen oder systemischen Kontrazeptiva beeinträchtigt werden und zusätzliche Empfängnisverhütungsmethoden sollten angewendet werden.^22^ Im adjuvanten Setting sollte bei bestehender Schwangerschaft keine systemische Therapie begonnen werden. Wird eine Schwangerschaft während einer laufenden adjuvanten Behandlung festgestellt, wird in der Regel ein Therapieabbruch der adjuvanten Systemtherapie empfohlen. Bei fortgeschrittener oder metastasierter Erkrankung kann eine systemische Therapie während der Schwangerschaft in Ausnahmefällen und nach strenger individueller Risiko‐Nutzen‐Abwägung erfolgen. Solche Entscheidungen sollten interdisziplinär in spezialisierten Tumorboards getroffen werden. Nach abgeschlossener Krebstherapie ist eine Schwangerschaft grundsätzlich möglich und nicht generell kontraindiziert. Allerdings kann ein erhöhtes Risiko für Schwangerschafts‐ und Geburtskomplikationen, insbesondere bei kurzem Abstand zwischen Therapieende und Konzeption bestehen. Eine engmaschige gynäkologische und onkologische Betreuung wird daher empfohlen. Zudem besteht nach Eintritt einer Schwangerschaft ein erhöhtes Rezidivrisiko sowie potenzielle Einschränkungen hinsichtlich Diagnostik und Therapie. Aus onkologischer Sicht sollte daher eine Beratung zum individuellen Rezidivrisiko und zum optimalen Zeitpunkt einer Schwangerschaft erfolgen.^31^, ^32^

## LEBENSQUALITÄT (QOL) ALS ZENTRALES VERSORGUNGSZIEL BEI MELANOMPATIENTEN

Die QoL ist ein zentrales Versorgungsziel in der onkologischen Versorgung, nicht nur im Kontext von Überleben, sondern als integraler Bestandteil der ganzheitlichen Patientenbetreuung. Sie geht über rein medizinische Endpunkte hinaus und umfasst wesentliche physische, psychische und soziale Dimensionen und die gesundheitsbezogene QoL (HRQoL).^33^ Während im vorangegangenen Abschnitt chronische therapieassoziierte Toxizitäten und Spätfolgen adressiert wurden, nimmt dieses Kapitel bewusst die subjektive Perspektive der Betroffenen ein. HRQoL umfasst sowohl körperliche als auch psychische, soziale und funktionelle Aspekte, die das tägliche Leben der Betroffenen prägen.^34^

Die QoL von Melanom‐Überlebenden umfasst physische, psychische, soziale und funktionelle Dimensionen.

Die systematische Erfassung der QoL ist von zentraler Bedeutung für die klinische Entscheidungsfindung, da sie eine differenzierte Abwägung zwischen therapeutischem Nutzen und potentiellen behandlungsassoziierten Belastungen ermöglicht. Für Melanompatienten gilt dies besonders, da operative und systemische Behandlungen Spätfolgen bis in die Survivorship‐Phase hinein erzeugen können.^12^, ^35^

Die systematische Erfassung der QoL ist für klinische Entscheidungen, Patientenberatung und Nachsorgeplanung essenziell.

### Erfassung der Lebensqualität (QoL): Instrumente und Methoden

Die QoL von Melanom‐Survivorn lässt sich mit unterschiedlichen Patient‐Reported Outcome (PRO)‐Instrumenten erfassen. Grundsätzlich werden allgemeine (generische) Fragebögen (Instrumente), die krankheitsunabhängig eingesetzt werden können (Tabelle 2),^36^, ^37^, ^38^ von HRQoL‐Spezifischen unterschieden, die gezielt tumorassoziierte Belastungen und therapiebedingte Effekte abbilden (Tabelle 3).^39^, ^40^, ^41^, ^42^ Für die klinische Praxis und wissenschaftliche Studien empfiehlt sich häufig eine Kombination beider Ansätze, um sowohl Vergleichbarkeit mit der Allgemeinbevölkerung als auch krankheitsspezifische Aspekte abzudecken. Dabei kommt in der Regel zunächst ein generisches Instrument zum Einsatz, das eine Vergleichbarkeit zwischen verschiedenen Erkrankungen sowie gesundheitsökonomische Bewertungen ermöglicht. Dieses wird ergänzt durch ein onkologiespezifisches HRQoL‐Instrument und gegebenenfalls durch das PRO‐CTCAE, um therapieassoziierte Spätfolgen gezielt zu erfassen. Dadurch lassen sich sowohl allgemeine als auch melanomspezifische QoL‐Effekte valide und klinisch relevant abbilden.

**TABELLE 2:** Allgemeine (generische) Instrumente zur Erfassung der Lebensqualität. Allgemeine (generische) Instrumente erfassen die gesundheitsbezogene QoL unabhängig von einer bestimmten Erkrankung und ermöglichen Vergleiche mit anderen Patientengruppen oder der Allgemeinbevölkerung.

Instrument	Beschreibung
**SF‐12 / VR‐12** (*Short Form / Veterans Rand Health Survey*)	Jeweils zwölf Fragen lange, vergleichbare Bögen, der physische und psychische Gesundheitsdimensionen (u. a. körperliche Funktion, Vitalität, soziale Funktion, psychisches Wohlbefinden) erfassen (lizenzpflichtig bzw. linzenzfrei).^36^
**EQ‐5D‐3L / EQ‐5D‐5L** (EuroQol)	Ein Kurzfragebogen mit fünf Kernbereichen (Mobilität, Selbstversorgung, Alltagsaktivitäten, Schmerzen/Unbehagen, Angst/Depression) und einer visuellen Analogskala, der die Berechnung sogenannter Utility‐Werte, also zusammenfassender Kennzahlen für den Gesundheitszustand des Patienten (0 = Tod, 1 = volle Gesundheit) ermöglicht, die klinische Veränderungen der Lebensqualität quantifizierbar und vergleichbar machen (genehmigungspflichtig, für akademische, nicht‐kommerzielle Nutzung meist gebührenfrei).^37^
**WHO‐5 Well‐Being Index**	Sehr kurzes generisches Instrument zur Erfassung des subjektiven psychischen Wohlbefindens; sensitiv für emotionale Belastungen (lizenzfrei für Forschung und klinische Anwendung, frei zugänglich, Quellenangabe nötig).^89^
**PROMIS‐29** (*Patient‐Reported Outcomes Measurement Information System* – 29)	Es misst mit sieben Kernbereichen verschiedene Aspekte der körperlichen, psychischen und sozialen Gesundheit auf einer gemeinsamen, standardisierten Skala, und ermöglicht so einen Vergleich zwischen Patientengruppen. Erweiterte Versionen sind vorhanden (lizenzpflichtig).^38^

**TABELLE 3:** Onkologie‐ und HRQoL‐spezifische Instrumente zur Erfassung der Lebensqualität. Die Tabelle fasst relevante Instrumente zur differenzierten Erfassung krankheitsspezifischer und therapieassoziierter Effekte im Survivorship zusammen. Folgende Abkürzungen wurden angewandt und sind nicht in der Tabelle aufgeführt gesundheitsbezogene Lebensqualität (HRQoL), Immuncheckpoint‐Inhibitor (ICI), Lebensqualität (QoL), QLQ‐SURV = *Quality of Life Questionnaire Survivorship*.

Instrument	Beschreibung
**EORTC QLQ‐C30** (European Organisation for Research and Treatment of Cancer – *Quality of Life Questionnaire – Core* 30)	Eines der am häufigsten eingesetzten onkologischen HRQoL‐Instrumente, das in den 1980er‐Jahren entwickelt wurde und 30 Items umfasst.; umfasst funktionelle Skalen (körperlich, emotional, sozial u. a.), Symptomskalen sowie globale QoL‐Bewertung (genehmigungspflichtig, für akademische, nicht‐kommerzielle Nutzung gebührenfrei).^39^
**EORTC *Survivorship*‐Module** (z. B. QLQ‐SURV)	Ergänzende Module zur spezifischen Erfassung von Langzeitfolgen und *Survivorship*‐Belastungen (analog zum QLQ‐C30 genehmigungspflichtig).^40^
**FACT‐M** (Functional Assessment of Cancer Therapy – Melanoma)	27 Fragen, die funktionelle Skalen umfassen, sowie 16 Fragen eines Melanom‐spezifischen Moduls, welches z. B. hautbezogene Beschwerden, funktionelle Einschränkungen, Angst vor Rezidiv, erfasst (genehmigungspflichtig, für akademische, nicht‐kommerzielle Nutzung meist gebührenfrei).^41^
**FACT‐ICM** (*Immune Checkpoint Modulators*):	Ein neues, speziell für Patienten mit ICI‐Therapie entwickeltes Instrument. Statt des Melanom‐Moduls werden die allgemeinen Fragen ergänzt um 25 Fragen zu spezifischen irAEs, z. B. Hautreaktionen, Gelenkbeschwerden oder gastrointestinale Symptome, die oft in der *Survivorship*‐Phase relevant bleiben.^42^
**PRO‐CTCAE** (*Patient‐Reported Outcomes – Common Terminology Criteria for Adverse Events*)	Ein spezifisches Instrument zur patientenberichteten Erfassung therapieassoziierter Nebenwirkungen (z. B. Fatigue, Gelenk‐/Muskelschmerzen, Hautsymptome), analog der CTCAE‐Einteilung von Nebenwirkungen durch ärztliches Personal, und damit besonders geeignet zur Ergänzung klassischer QoL‐Instrumente bei Immuntherapie‐ und *Survivorship*‐Fragestellungen (lizenzfrei, frei zugänglich, Quellenangabe nötig).^90^

Für *Survivorship*‐Fragestellungen empfiehlt sich eine Kombination aus generischen und onkologischen HRQoL‐Instrumenten.

Die Erhebung der QoL erfolgt in der Regel als patientenberichtetes Outcome (PRO) papierbasiert oder zunehmend elektronisch (ePRO). Die Erhebungsfrequenz sollte sich an Therapiesituation und Nachsorgephase orientieren: vor Beginn einer systemischen Therapie als Baseline, während der Behandlung in regelmäßigen Abständen (zum Beispiel alle 4–12 Wochen) sowie in der *Survivorship*‐Phase in größeren Intervallen, etwa halbjährlich oder jährlich, ergänzt durch anlassbezogene Erhebungen bei neu auftretenden Beschwerden oder Therapiewechseln. Ziel ist es, relevante Veränderungen frühzeitig zu erkennen und supportive Maßnahmen gezielt einzuleiten.

### Studienlage zur Lebensqualität bei Melanom‐Überlebenden

Erkenntnisse zur HRQoL von Melanom‐Überlebenden haben in den letzten Jahren zugenommen, bleiben jedoch heterogen hinsichtlich Populationen, Messinstrumenten und Nachbeobachtungsdauer.^35^, ^43^, ^44^, ^45^, ^46^, ^47^ Der Schwerpunkt der verfügbaren Daten liegt weniger auf einzelnen therapieassoziierten Nebenwirkungen, sondern auf funktionellen, psychosozialen und alltagsrelevanten Dimensionen der QoL im Langzeitverlauf. Mehrere populationsbasierte Querschnittsstudien zeigen, dass viele Melanom‐Langzeitüberlebende im globalen HRQoL‐Score Werte erreichen, die denen der alters‐ und geschlechtsangepassten Allgemeinbevölkerung entsprechen. Insbesondere bei Patienten nach frühen Stadien oder nach erfolgreicher Primärtherapie unterscheiden sich generische Lebensqualitätsmaße häufig nur geringfügig von Referenzkollektiven.^48^, ^49^ Gleichzeitig weisen differenzierte Subskalenanalysen darauf hin, dass einzelne Funktionsbereiche (zum Beispiel körperliche Rollenfunktion, Vitalität oder soziale Teilhabe) auch Jahre nach Diagnose noch eingeschränkt sein können, ohne dass sich dies zwingend in der globalen Lebensqualitätsbewertung widerspiegelt.^50^, ^51^ Dies unterstreicht die Bedeutung multidimensionaler Instrumente.#

Subskalenanalysen zeigen, dass einzelne Funktionsbereiche wie Rollenfunktion, Vitalität oder soziale Teilhabe auch Jahre nach Diagnose eingeschränkt sein können.

### Psychosoziale Dimensionen und Krankheitsverarbeitung

Unabhängig von der Tumorkontrolle berichten viele Überlebende über anhaltende psychosoziale Herausforderungen. Besonders häufig werden ausgeprägte Angst vor einem Rezidiv,^52^, ^53^ veränderte Selbstwahrnehmung und beeinträchtigtes Körperbild,^54^ sowie Unsicherheiten bei der beruflichen und sozialen Reintegration beschrieben.^54^, ^55^, ^56^, ^57^ Darüber hinaus bestehen bei manchen Betroffenen emotionale Belastungen fort, selbst bei objektiv günstiger Prognose.^56^, ^58^ Studien zeigen, dass ein relevanter Anteil der Betroffenen klinisch relevante Angstsymptome oder reduzierte emotionale Funktion angibt, auch wenn die körperliche Leistungsfähigkeit weitgehend erhalten ist. Weibliches Geschlecht, jüngeres Alter bei Diagnose sowie frühere Rezidive sind dabei mit geringerer HRQoL assoziiert.^43^, ^53^, ^58^

Weibliches Geschlecht, jüngeres Alter bei Diagnose und frühere Rezidive sind mit geringerer HRQoL assoziiert.

Diese Befunde verdeutlichen, dass die *Survivorship*‐Phase nicht automatisch mit psychischer Entlastung gleichzusetzen ist, sondern eine eigenständige Anpassungs‐ und Verarbeitungsphase darstellt. Neben der emotionalen Dimension rücken funktionelle Aspekte in den Fokus: Die Rückkehr in den Beruf, die Wiederaufnahme sozialer Rollen und die subjektive Leistungsfähigkeit im Alltag stellen zentrale Einflussfaktoren der QoL dar. Langzeitstudien zeigen, dass viele Melanom‐Überlebende zwar ihre grundlegende Autonomie bewahren, jedoch Einschränkungen in Rollenfunktionen, sozialer Aktivität oder Vitalität berichten.^43^, ^59^, ^60^ Damit wird HRQoL zunehmend als Ausdruck einer funktionellen und psychosozialen Adaptation verstanden und nicht nur als Abwesenheit medizinischer Probleme, mitunter bleiben einzelne Funktions‐ und Belastungsbereiche dauerhaft beeinträchtigt.^35^, ^43^ Zu den Einflussfaktoren einer eingeschränkten HRQoL in der *Survivorship*‐Phase zählen unter anderem ein fortgeschrittenes Tumorstadium zum Zeitpunkt der Erstdiagnose,^58^ relevante Komorbidität,^60^ sowie psychosoziale Belastungsfaktoren,^53^ und eine unzureichende soziale Unterstützung.^43^, ^60^ Da globale QoL Scores diese Heterogenität häufig nur unvollständig abbilden, ist der Einsatz spezifischer Subskalen sowie ergänzender psychosozialer Screeninginstrumente angezeigt.

## BESCHWERDEN UND BEDÜRFNISSE LANGZEITÜBERLEBENDER MELANOMPATIENTEN

### Lebensqualität (QoL) im *Survivorship*: Ergebnisse aktueller Querschnittsstudien

Die klinische Relevanz chronischer Nebenwirkungen liegt weniger in der Mortalität als vielmehr in der nachhaltigen Beeinträchtigung der QoL. In Survivorship‐Kohorten sind chronische Fatigue, gastrointestinale Beschwerden und muskuloskelettale Schmerzen eng mit einer reduzierten körperlichen und psychosozialen QoL assoziiert.^61^, ^62^, ^63^ Robert et al. zeigten zudem, dass diese Symptome die berufliche Wiedereingliederung und soziale Teilhabe deutlich beeinträchtigen können.^12^, ^64^ Mehrere *Real‐World*‐Kohorten liefern hierzu detaillierte Einblicke in die derzeitige Versorgungssituation.^12^, ^35^, ^65^ In einer Querschnittsbefragung von Langzeitüberlebenden (≥ 5 Jahre nach Eintritt in Stadium IV) zeigte sich eine hohe Prävalenz anhaltender behandlungsassoziierter Beschwerden. Etwa 46 % der Patienten, die mindestens eine Systemtherapie erhalten hatten, berichteten über persistierende körperliche Symptome, die sie einer früheren Systemtherapie zuschrieben.^12^ Aber auch operative und strahlentherapeutische Interventionen führen bei einem relevanten Anteil der Patienten zu persistierenden Langzeitfolgen. Nach operativen Eingriffen berichteten etwa 42 % der Patienten über fortbestehende Einschränkungen (zum Beispiel Taubheitsgefühle, Bewegungseinschränkungen oder Lymphödeme). Nach Radiotherapie gaben knapp 44 % der bestrahlten Patienten persistierende Beschwerden an, darunter funktionelle Limitationen und Sensibilitätsstörungen; nach zerebraler Bestrahlung traten zudem kognitive Beeinträchtigungen, insbesondere Störungen von Gedächtnis und Konzentration, in den Vordergrund.^12^

In einer multizentrischen Querschnittsstudie von Patienten mit Melanom im Stadium IV nach Behandlung mit Pembrolizumab konnte nach medianer Nachbeobachtung von 9,1 Jahren gezeigt werden, dass sich das subjektive psychische Wohlbefinden nicht signifikant zwischen den befragten Melanompatienten, auch nicht zwischen denjenigen mit vorausgegangenen irAE, und altersstratifizierten Referenzwerten der Allgemeinbevölkerung unterschied. Bei Patienten mit persistierenden Symptomen fanden sich lediglich in der kleinen Subgruppe der 41‐ bis 60‐Jährigen signifikant schlechtere Werte. Überlebende mit irAE während der Pembrolizumab‐Therapie berichteten jedoch insgesamt über signifikant niedrigere QoL in der Nachbeobachtung im Vergleich zu Patienten ohne irAE. Ein ähnlicher jedoch nicht signifikanter Trend zeigte sich bei Langzeitüberlebenden mit persistierenden Symptomen (p = 0,17).^35^ Die rollenbezogene Funktionsfähigkeit wurde mithilfe der Rollenfunktionsskala (*Role Functioning Scale*, RFS) erhoben, die das Funktionsniveau in den Bereichen Arbeit/Produktivität, selbstständiges Leben und Selbstversorgung, sowie Beziehungen im unmittelbaren und erweiterten sozialen Umfeld erfasst.^35^, ^66^ Bei Langzeitüberlebenden zeigte sich im Vergleich zur Allgemeinbevölkerung eine niedrigere Ausprägung (p = 0,08).^35^

### Psychoonkologische Belastung und emotionaler Bedarf in Realität und Praxis

Bei Stadium‐IV‐Langzeitüberlebenden zeigten etablierte Screening‐Instrumente zur Erfassung von psychoonkologischen Belastung (Distress Thermometer, Hornheide Screening Instrument), dass etwa ein Drittel der Befragten Werte oberhalb des Grenzwertes erreichte und somit formal psychoonkologische Unterstützung benötigt.^12^ Auffällig ist in diesem Zusammenhang die immer wieder feststellbare Diskrepanz zwischen dem objektiv durch Screening‐Instrumente erfassten psychoonkologischen Unterstützungsbedarf und der subjektiven Selbsteinschätzung der Patienten. Deutlich weniger Patienten gaben in ihrer Selbsteinschätzung an, psychoonkologische Hilfe zu benötigen, als es die Screeninginstrumente nahelegten.^12^ Dieser Aspekt ist für die klinische Praxis relevant, da er möglicherweise auf eine Hemmschwelle gegenüber der Inanspruchnahme psychosozialer Hilfe hinweist, eventuell aus Sorge vor Stigmatisierung. Sicherlich gibt es auch Patienten, die zwar belastet sind, jedoch über ausreichend soziale Kontakte / Ressourcen verfügen, sodass zum Erhebungszeitpunkt kein weiterer Unterstützungsbedarf besteht.^67^ Da diese Ressourcen und die Belastungssituation dynamisch sind und sich im zeitlichen Verlauf verändern können, ist ein regelmäßiges Rescreening, beispielsweise im Rahmen von *Survivorship*‐Programmen indiziert und notwendig. Auch Nalabothu und Johnson heben die Bedeutung strukturierter Nachsorgeprogramme und interdisziplinärer *Survivorship*‐Konzepte hervor. Diese sollten zudem regelmäßige endokrinologische Kontrollen, neurologische Verlaufskontrollen sowie die systematische Erfassung patientenberichteter Symptome beinhalten.^68^

Deutlich weniger Patienten gaben in ihrer Selbsteinschätzung an, psychoonkologische Hilfe zu benötigen, als es die Screeninginstrumente nahelegten.

In der MESSAGE‐Studie konnte innerhalb der untersuchten Stadium‐III‐Kohorte kein Zusammenhang zwischen Therapieverfahren oder Überlebensdauer und der psychischen Belastung festgestellt werden. Eine vermutete Assoziation zwischen Krankheitsparametern und langfristigem Wohlbefinden konnte damit nicht bestätigt werden.^65^ Dagegen bestand bei Patienten im Stadium IV eine signifikante Verbindung zwischen erhöhter psychosozialer Belastung und persistierenden Nebenwirkungen systemischer Therapien.^12^ Bei Stadium‐III‐Überlebenden war der mittlere Distress moderat, jedoch lagen die Werte häufig über gängigen Grenzwerten. Depressions‐ und Angstscores waren im Vergleich zur Allgemeinbevölkerung erhöht, während die globale Lebensqualität insgesamt vergleichbar blieb.^65^ Das unterstreicht ein häufiges *Survivorship*‐Paradox: Patienten bewerten ihre Lebensqualität selbst als insgesamt gut, während gleichzeitig eine relevante psychische Symptombelastung und krankheitsbezogene Alltagslast fortbestehen.

## NACHSORGE UND THERAPIEPLANUNG NACH KOMPLETTREMISSION UNTER IMMUNTHERAPIE

### Versorgungsrealität nach Komplettremission unter Immuntherapie

**ABBILDUNG 3:**
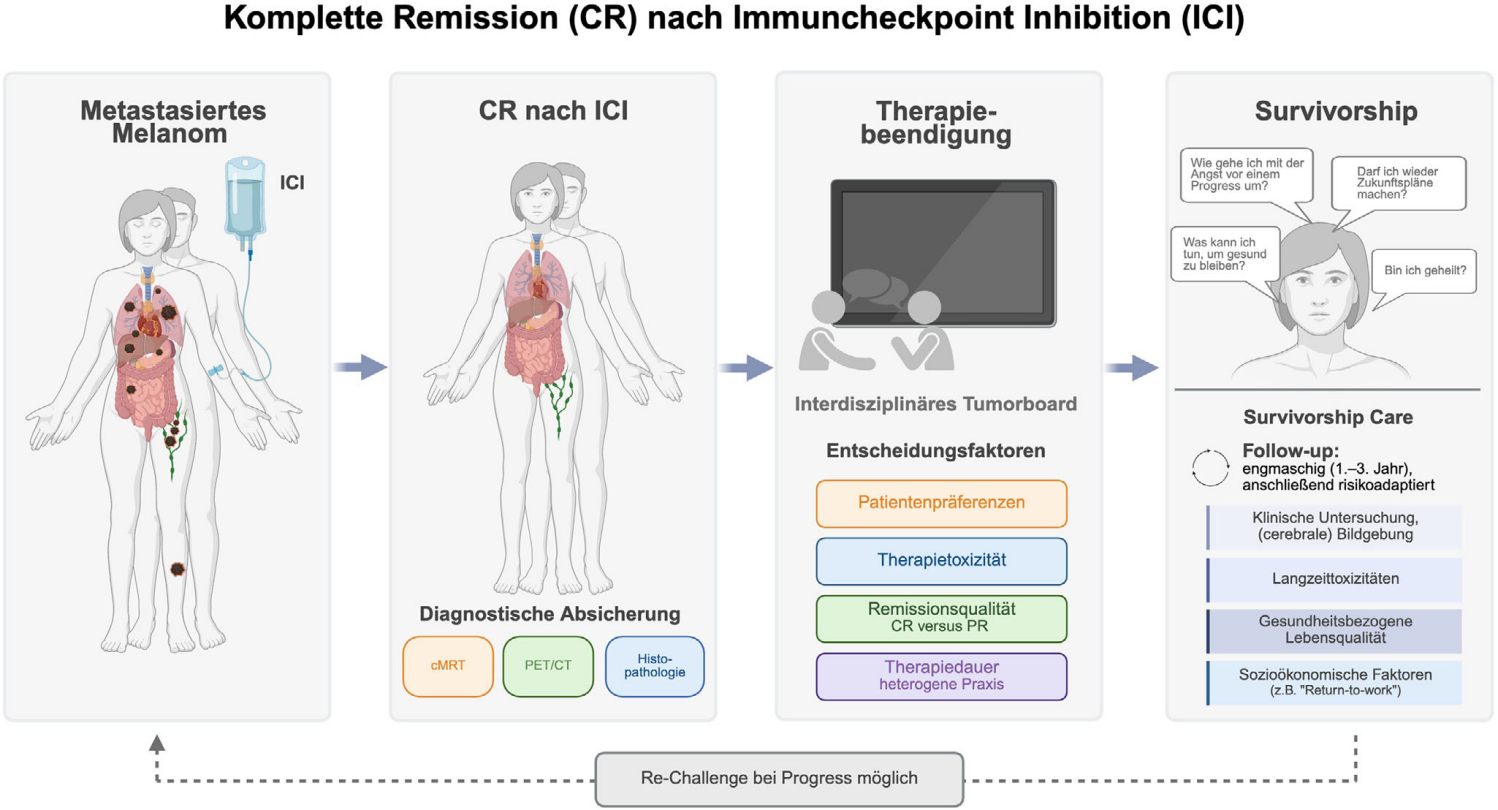
Nachsorge und Therapieplanung nach Komplettremission (CR) unter Immuntherapie (ICI). Schematische Darstellung der Nachsorge bei Patienten mit Melanom nach Erreichen einer Komplettremission (CR) im klinischen Alltag. Die Abbildung wurde mithilfe der Software BioRender (BioRender.com, Toronto, Kanada) erstellt.

Mit der zunehmenden Etablierung von ICI hat sich das Behandlungsspektrum des metastasierten Melanoms grundlegend verändert. Ein relevanter Anteil der Patienten erreicht heute unter ICI eine CR, wodurch sich die klinische Fragestellung zunehmend von der Akuttherapie hin zum Management nach Remission verschiebt (Abbildung 3). Eine Umfrage unter 51 deutschsprachigen Hautkrebszentren (Deutschland, Schweiz, Österreich) stellt die bislang umfassendste Erhebung dar zum konkreten Vorgehen im Alltag nach Erreichen einer CR mit ICI und bildet die aktuelle Versorgungssituation im deutschsprachigen Raum systematisch ab.^69^ In zwei Dritteln der Hautkrebszentren wurde die Therapie nach Erreichen einer CR unter ICI für bis zu sechs Monate fortgeführt und anschließend beendet. Das Absetzen der ICI nach erreichter CR stellt in der klinischen Praxis keineswegs eine Ausnahme dar. Gleichzeitig zeigte sich im Falle einer PR oder SD ein sehr heterogenes Vorgehen, das von wenigen Monaten bis zu mehr als zwei Jahren reichte. Die Entscheidungsfindung erfolgt überwiegend interdisziplinär im Tumorboard, was den komplexen Charakter dieser Fragestellung widerspiegelt. Auffällig ist jedoch der hohe Stellenwert der Patientenpräferenz: Therapieassoziierte Belastungen, kumulative Toxizitäten, organisatorischer Aufwand sowie der Wunsch nach Rückkehr in einen möglichst therapiefreien Alltag spielen eine zentrale Rolle bei der Äußerung des Wunsches nach einem vorzeitigen Therapieende. Die Initiierung der Diskussion einer Therapiepause ohne Anstoß durch die Patienten war sehr selten.

Vor der Beendigung der ICI wurde in der Mehrzahl der Zentren eine intensive diagnostische Absicherung der CR vorgenommen. Dazu zählten nahezu regelmäßig eine zerebrale Bildgebung (in 94 % ein MRT) und ein PET/CT (in 86 % der Zentren), sowie bei verbleibenden unklaren Befunden eine histologische Abklärung. Auch die Nachsorge nach Beendigung der ICI erwies sich als heterogen, insgesamt jedoch eher bildgebungsintensiv, insbesondere in den ersten ein bis drei Jahren. In vielen Zentren wurde die radiologische Nachsorge über fünf Jahre oder länger fortgeführt, teilweise unabhängig von individuellen Risikofaktoren. Es wurde deutlich, dass diese klinische Praxis auf einer begrenzten Evidenzbasis beruhte und durch eine erhebliche Variabilität gekennzeichnet war, was den dringenden Bedarf an prospektiven Daten unterstreicht.^69^

### Bedarf an und bisher durchgeführte prospektive Studien

Vor dem Hintergrund der derzeitigen Versorgungsrealität kommt der 2025 von van der Veldt et al. auf dem ESMO Kongress präsentierten Safe‐Stop‐Studie eine besondere Bedeutung zu. Sie ist die erste abgeschlossene prospektive Studie, die gezielt das frühe Absetzen einer anti‐PD‐1‐Therapie nach bestätigter CR oder PR untersucht hat. Damit unterscheidet sie sich grundlegend von bislang verfügbaren Daten, die überwiegend aus retrospektiven Kohorten, Experten‐Surveys (zum Beispiel. DeCOG/ADO) oder Zulassungsstudien mit definierter Behandlungsdauer von meist 24 Monaten stammen. Erstmals wurde damit prospektiv eine zentrale Survivorship‐Frage adressiert.

Insgesamt wurden 200 Patienten eingeschlossen, davon 155 mit Stadium IV. Zum Zeitpunkt des Therapieabbruchs hatten 58 Patienten (29 %) eine CR und 142 (71 %) eine PR erreicht. Alle erhielten eine Erstlinientherapie mit Nivolumab oder Pembrolizumab und hatten darunter eine CR oder PR erreicht. Die mediane Behandlungsdauer betrug lediglich 24 Wochen, bei einer medianen Nachbeobachtung von 54 Monaten. Innerhalb von 24 Monaten nach Beendigung der ICI entwickelten 33 von 200 Patienten (17 %) einen Progress. Neue Hirnmetastasen traten bei 9 Patienten (5 %) auf, und 15 Patienten (8 %) verstarben melanombedingt. Angesichts eines Stadium‐IV‐Kollektivs und einer medianen Therapiedauer von nur sechs Monaten sind diese Ereignisraten bemerkenswert niedrig. In der univariablen Analyse zeigte sich ein klarer prognostischer Unterschied zwischen CR und PR zum Zeitpunkt des Therapieabbruchs. Im Vergleich zur CR war eine PR mit einem signifikant erhöhten Progressionsrisiko assoziiert (HR 2,21; p = 0,02).^70^ Zusammenfassend zeigte sich in dieser Studie trotz des frühen Therapieabbruchs eine hohe Rate an anhaltenden Remissionen nach 24 Monaten. Diese Strategie erwies sich als nicht unterlegen gegenüber der historischen Kontrollkohorte aus der KEYNOTE‐006 Studie.^7^, ^70^, ^71^

Im Gegensatz zu diesen Daten wies eine 2025 veröffentlichte systemische Übersichtsarbeit inklusive einer Meta‐Analyse von 20 Studien mit insgesamt 1832 Patienten mit malignem Melanom auf ein höheres Rezidivrisiko bei kürzerer Therapiedauer hin. Demnach lag bei Patienten, die länger als zwei Jahre behandelt wurden, das 1‐Jahres‐ Progressionsfreie Überleben (PFS) bei 95 %. Bei einer Behandlungsdauer von 1–2 Jahren beziehungsweise weniger als einem Jahr reduzierten sich das 1‐Jahres‐PFS auf 91 % beziehungsweise 82 %. Entschieden sich die Patienten für eine elektive Therapieunterbrechung, war die 1‐Jahres‐PFS‐Rate im Vergleich zu der Gruppe mit therapiebedingten Abbrüchen aufgrund von Nebenwirkungen signifikant erhöht (91 % vs. 79 %).^72^ Für die *Survivorship*‐Beratung besonders relevant ist, dass ein Progress nach frühem Absetzen nicht das Ende therapeutischer Optionen bedeutet. Die Wiederaufnahme der ICI Therapie kann erneut eine Krankheitskontrolle erzielen.^73^, ^74^

Die Nachsorgeintervalle sollten in den ersten ein bis drei Jahren engmaschig erfolgen und anschließend risikoadaptiert verlängert warden. Der Fokus sollte hier auf der klinischen Beurteilung, zerebraler Bildgebung und Berücksichtigung der QoL liegen. In der *Survivorship*‐Situation rücken Fatigue, endokrine Langzeittoxizitäten und insbesonderedie Angst vor einem Rückfall in den Vordergrund der Betreuung.^75^ Gleichzeitig ist es wichtig zu vermitteln, dass ein Rezidiv nicht zwangsläufig eine prognostisch schlechte Situation bedeutet. In vielen Fällen kann erneut eine effektive Krankheitskontrolle erreicht werden.

Für die *Survivorship*‐Beratung besonders relevant ist, dass ein Progress nach frühem Absetzen nicht das Ende therapeutischer Optionen bedeutet. Viele Patienten konnten erneut erfolgreich mit ICI behandelt werden, teilweise in Kombination mit lokalen operativen oder stereotaktischen Eingriffen.

## *SURVIVORSHIP*‐CARE PROGRAMME: AUSBLICK UND STAND DER AKTUELLEN FORSCHUNG

Das von der Deutschen Krebshilfe geförderte Projekt „Leben mit Melanom“ (LeMela) widmet sich der Entwicklung und Erprobung eines *Survivorship‐Care*‐Programms für Menschen mit metastasiertem Melanom unter und nach Therapie. In einem im Rahmen des LeMeLa‐Programms erstellten *Scoping‐Reviews* erfüllten sieben Studien (neun Artikel) die Einschlusskritieren und konnten in die Analyse eingeschlossen. Es konnten sechs *Survivorship‐Care*‐Konzepte und dazugehörige Komponenten herausgearheitet werden. Hierzu gehören die onkologische Nachsorge (in Bezug auf körperliche Funktionen),^76^, ^77^ die psychosoziale Nachsorge,^76^, ^77^, ^78^ die Überwachung auf Rezidive und neue Krebserkrankungen,^77^, ^79^, ^80^ Ressourcen und Koordination der Versorgung,^76^, ^77^ Information und Empowerment^76,77,81^: und sogenannte multimodale Ansätze (Tabelle 4).^82^, ^83^, ^84^

**TABELLE 4:** Survivorship‐Care‐Konzepte.

Survivorship‐Care‐Konzepte	Beschreibung
**Onkologische Nachsorge (in Bezug auf körperliche Funktionen)** ^76^, ^77^	Screening und Management akuter und chronischer, immunbedingter unerwünschter Ereignisse, Berücksichtigung der Auswirkungen auf die Fruchtbarkeit, Screening auf Endokrinopathien, Beurteilung von Lymphödemen, Screening auf Fatigue und Erwägung einer Überweisung in die Palliativmedizin.
**Psychosoziale Nachsorge** ^76^, ^77^, ^78^	Screening auf Ängste und Depressionen, Kartierung des lokaleren Zugangs zu Psychologen und Psychiatern, Einschätzung der sozialarbeiterischen Situation, und Screening auf finanzielle Belastungen, spirituell ausgerichtete Meditation,
**Überwachung auf Rezidive und neue Krebserkrankungen** ^77^, ^79^, ^80^	Haut‐ und Lymphknotenuntersuchungen, routinemäßige Bluttests und Bildgebung sowie eine digitale, tabletbasierte Intervention zur Unterstützung der vollständigen Selbstuntersuchung.
**Ressourcen und Koordination der Versorgung** ^76^, ^77^	*Survivorship‐Care*‐Plan, und multidisziplinäre Zusammenarbeit und feste Ansprechpartner.
**Information und *Empowerment*** **76** **^,^** **77** **^,^** **81**	Personalisierte Informationen/Beratung, u. a. über Risikofaktoren und die Bereitstellung von Informationen über zuverlässige Quellen, von Peers geleitete soziale Unterstützung per Telefon
**Multimodaler Ansatz** ^82^, ^83^, ^84^	*Model of nurse‐led, telehealth‐delivered survivorship care* (MELCARE) und die *Melanoma Care Study*.

Die identifizierten *Survivorship‐Care*‐Konzepte zeichnen sich häufig durch einen multidisziplinären Ansatz aus. Drei der sieben Studien bezogen sich auf Menschen mit einem Melanom im Stadium III–IV.^76^, ^78^, ^82^ Zwei weitere Studien gaben keine detaillierten Angaben zur Zielgruppe,^77^, ^81^ während sich zwei Studien auf Patienten mit Melanomen im Stadium 0–IIc konzentrierten.^79^, ^80^, ^83^, ^84^ Die Umsetzung der identifizierten *Survivorship‐Care*‐Konzepte erfordert erhebliche Investitionen in personelle und technologische Ressourcen.^78^, ^79^, ^80^, ^82^, ^84^ Dennoch scheinen einzelne Konzepte, wie die psychosoziale Nachsorge und die Überwachung auf Rezidive und neue Krebserkrankungen, Ängste und depressive Symptome zu reduzieren,^83^ und die QoL zu verbessern.^79^ Es besteht eine Lücke in der empirischen Forschung zur Gesundheitskompetenz im Kontext von *Survivorship Care*. Maßgeschneiderte Informationen und bedarfsorientierte Interventionen, die auch individuelle Risiken für potenzielle negative medizinische und psychosoziale Folgen berücksichtigen, sind Schlüsselkomponenten bei der Umsetzung von *Survivorship‐Care*‐Plänen für Krebspatienten im Allgemeinen.^85^ Angesichts der Fülle verfügbarer Gesundheitsinformationen und der zunehmenden Nutzung digitaler Gesundheitsanwendungen sollte die Förderung der Gesundheitskompetenz von Patienten eine hohe Priorität haben.^85^ Darüber hinaus fehlen gesundheitsökonomische Analysen. Um die Wirksamkeit von *Survivorship‐Care*‐Konzepten zu untersuchen, sind weitere Studien erforderlich. Die Ergebnisse werden die Entwicklung eines patientenzentrierten *Survivorship‐Care*‐Programms für Menschen mit Melanomen unterstützen.

## REHABILITATION

**ABBILDUNG 4:**
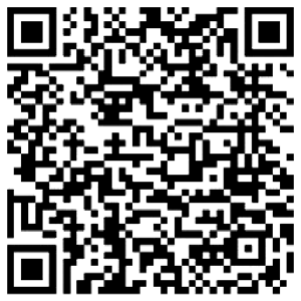
QR Code, Das Rehaportal

Die S3‐Leitlinie Melanom betont, dass die Nachsorge neben der Tumorkontrolle auch funktionelle, psychosoziale und soziale Folgen der Erkrankung berücksichtigen sollte. In diesem Kontext wird die onkologische Rehabilitation als Bestandteil eines biopsychosozialen Versorgungskonzeptes genannt, insbesondere zur Behandlung von Fatigue, psychischer Belastung sowie Einschränkungen der Alltags‐ und Erwerbsteilhabe.^21^ Alle Patienten in Deutschland haben grundsätzlich einen gesetzlichen Anspruch auf medizinische Rehabilitation mit dem Ziel, die Teilhabe am sozialen und beruflichen Leben wiederherzustellen oder zu sichern (§ 40 SGB V, § 9 SGB VI, § 31 SGB XI). Im Rahmen des Wunsch‐ und Wahlrechts kann die geeignete Rehabilitationseinrichtung selbst gewählt werden (§ 8 SGB IX). Eine (dermato)onkologische Rehabilitation kann nach kurativ intendierter Therapie, während einer Erhaltungstherapie sowie auch in der palliativen Situation beantragt werden. Eine Übersicht potenziell geeigneter Rehabilitationseinrichtungen für das Krankheitsbild C43 (bösartiges Melanom der Haut) ist über folgendes Online‐Portal zugänglich (siehe CR Code, Abbildung 4):

Voraussetzung sind eine ausreichende Motivation, Belastbarkeit und Selbstständigkeit der Patienten (Reha‐Fähigkeit) sowie ein festgestellter Reha‐Bedarf im Sinne gesundheitlicher Einschränkungen mit realistischer Aussicht auf Besserung. Bei fortbestehenden Funktionseinschränkungen besteht die Möglichkeit weitere Reha‐Maßnahmen zu beantragen (https://www.deutsche‐rentenversicherung.de/DRV/DE/Reha/Reha‐Antragstellung/reha‐antragstellung_node.html; www.reha‐hilft‐krebspatienten.de). Auch wenn spezifische randomisierte Rehabilitationsstudien bei Melanom‐Überlebenden bislang begrenzt sind, ist der Nutzen onkologischer Rehabilitation insgesamt gut belegt. Aktuelle Metaanalysen zeigen, dass bewegungsbasierte und multimodale Rehabilitationsprogramme bei Krebspatienten zu signifikanten Verbesserungen von Fatigue, körperlicher Leistungsfähigkeit, HRQoL, sowie Angst und Depression führen. Diese Effekte betreffen zentrale Problembereiche des Melanom‐*Survivorships* und stützen die Übertragbarkeit rehabilitativer Konzepte auf diese Patientengruppe.^86^, ^87^, ^88^ Vor diesem Hintergrund sollte die onkologische Rehabilitation frühzeitig in die Nachsorgeplanung von Melanom‐Patienten integriert werden. Dermatologen kommt dabei eine Schlüsselrolle zu, da sie Patienten im Rahmen der Nachsorgeprogramme langfristig begleiten.

Alle Patienten in Deutschland haben grundsätzlich einen gesetzlichen Anspruch auf medizinische Rehabilitation mit dem Ziel, die Teilhabe am sozialen und beruflichen Leben wiederherzustellen oder zu sichern (§ 40 SGB V, § 9 SGB VI, § 31 SGB XI).

## ZUSAMMENFASSUNG UND AUSBLICK

Nachdem über Jahrzehnte hinweg die Prognose des metastasierten Melanoms infaust war, konnte durch die Einführung der ICI eine drastische Verbesserung des Gesamtüberlebens erreicht werden. Die Langzeitüberlebenden sind mittlerweise in unseren Sprechstunden angekommen und stellen den Behandler vor neue Herausforderungen. Insbesondere chronische immunvermittelte Toxizitäten, die Rückkehr in das „alte Leben“ und der berufliche Wiedereinstieg fordern Patienten und Behandler. Vor dem Hintergrund eines nun potentiell erreichbaren, langzeitigen Überlebens trotz vormals vorhandener nicht‐resezierbarer Metastasen, muss die Therapieplanung von Beginn an diesen Aspekt mit berücksichtigen. Die Patienten sind aufgrund der verbesserten Therapiemöglichkeiten mittlerweile grundsätzlich in der Lage, Langzeittoxizitäten, beispielsweis nach einer Radiatio, noch zu erleben. Der Stellenwert von Rehabilitationsmaßnahmen und *Survivorship*‐Programmen ist daher hoch und neue Forschungsbereiche tun sich auf. Innerhalb der ADO wurde ein Komitee „*Survivorship*“ gegründet und in Leipzig wurde 2025 eine neue Professur „*Cancer Survivorship*“ eingerichtet (Stand 02/2026). Dennoch bedarf es für das Melanom der Etablierung flächendeckender *Survivorship‐Care* Programme wie beispielsweise beim Mammakarzinom. Die Implementierung telemedizinischer Sprechstunden oder App‐basierter Nachsorgeprogramme könnte hierfür hilfreich sein. Interdisziplinäre Zusammenarbeit ist dabei sicherlich der Schlüssel dafür, Langzeitüberlebende optimal zu betreuen.

## DANKSAGUNG

Open access Veröffentlichung ermöglicht und organisiert durch Projekt DEAL.

## INTERESSENKONFLIKT

MR erhielt eine Förderung im Rahmen des Junior Clinician Scientists Program der Universität Tübingen (Antragsnummer 523‐0‐0) sowie Reisekostenzuschüsse von Almirall Hermal, Galderma und Pierre Fabre, die alle nicht im Zusammenhang mit der eingereichten Arbeit standen.

SDB erhielt ein Honorar für einen Vortrag von der Medea GmbH, das nicht im Zusammenhang mit der eingereichten Arbeit stand.

CLB erhielt eine Förderung im Rahmen des Mildred Scheel Nachwuchszentrums (MSNZ) des Universitätsklinikums Hamburg‐Eppendorf (UKE), finanziert durch die Deutsche Krebshilfe, die nicht im Zusammenhang mit der eingereichten Arbeit steht.

EL berichtet über eine beratende Tätigkeit oder hat Honorare/Reisekosten erhalten von: Bristol‐Myers Squibb, Merck Sharp & Dohme, Kyowa Kirin, Novartis, Pierre Fabre, Regeneron, Sanofi, Sunpharma und Takeda.

MS berichtet über eine beratende Tätigkeit oder hat Honorare/Reisekosten erhalten von: Bristol‐Myers Squibb, Merck Sharp & Dohme, Immunocore, Novartis, Pierre Fabre, Regeneron und Sunpharma, allesamt außerhalb der eingereichten Arbeit

MVH erhielt Beratungshonorare von Novartis, Immunocore, BMS, MSD, Sanofi, Almirall, Pierre Fabre, Infectopharm und Galderma, außerhalb der eingereichten Arbeit.

GS war als Berater für Novartis tätig, außerhalb der eingereichten Arbeit.

FM war als Berater tätig und/oder hat Honorare von Novartis, Bristol‐Myers Squibb, Merck Sharp & Dohme, Pierre Fabre, Sanofi Genzyme und Sun Pharma sowie Reisekostenzuschüsse von Novartis, Sun Pharma, Pierre Fabre und Merck Sharp & Dohme erhalten, außerhalb der eingereichten Arbeit.

LZ berichtet über eine beratende Tätigkeit oder hat Honorare/Reisekosten von folgenden Unternehmen erhalten: Bristol‐Myers Squibb, Merck Sharp & Dohme, Novartis, Pierre Fabre, Regeneron, Sanofi und Sunpharma.

AF war als Berater für Novartis, MSD, BMS, Pierre‐Fabre und Immunocore tätig; erhielt Reisekostenzuschüsse von Novartis, BMS und Pierre‐Fabre sowie Vortragshonorare von Novartis, BMS und MSD und gibt institutionelle Forschungszuschüsse von der BMS Stiftung Immunonkologie an, die nicht im Zusammenhang mit der eingereichten Arbeit stehen.

Die übrigen Autoren erklären, dass sie keine Interessenkonflikte haben.

## [CME QUESTIONS – LERNERFOLGSKONTROLLE]

1. Welche Aussage zur Definition des „*Cancer Survivor*“ ist korrekt?
  - Als *Cancer Survivor* gelten ausschließlich Patienten, die mindestens fünf Jahre nach Krebsdiagnose überlebt haben.
  - Ein *Cancer Survivor* ist jeder Mensch ab Abschluss der Krebstherapie bis zum Lebensende.
  - Der Begriff *Cancer Survivor* beschreibt nur Patienten ohne aktive Tumorerkrankung.
  - *Cancer Survivor* sind ausschließlich Patienten mit kompletter Remission nach einer Krebserkrankung.
  - Ein *Cancer Survivor* ist jeder Mensch vom Zeitpunkt der Krebsdiagnose bis zum Lebensende – unabhängig von Krankheitsstadium oder Verlauf.
2. Wie definiert die *Society for Immunotherapy of Cancer (SITC)* ein chronisches immunvermitteltes unerwünschtes Ereignis (chronisches irAE)?
  - Nebenwirkungen, die 3 Monate nach Absetzen der ICI‐Therapie persistieren
  - Nebenwirkungen, die während der laufenden Immuntherapie auftreten
  - Nebenwirkungen, die länger als 6 Monate nach Krebsdiagnose bestehen
  - Nebenwirkungen, die ausschließlich eine immunsuppressive Therapie erfordern
  - Nebenwirkungen, die erst mehr als 12 Monate nach Therapieende auftreten
3. Welche Aussage zu endokrinen Langzeittoxizitäten unter Immuncheckpoint‐Inhibitoren beim Melanom ist korrekt?
  - Endokrine irAEs treten selten auf und sind meist vollständig reversibel.
  - Endokrine Nebenwirkungen entstehen ausschließlich spät nach Therapieende.
  - Hypothyreose und Hypophysitis sind häufig irreversibel und erfordern meist eine lebenslange Hormonsubstitution.
  - Endokrine irAEs verursachen keine langfristigen funktionellen Einschränkungen.
  - Eine Verlaufskontrolle ist nach Therapieende nicht erforderlich.
4. Welche Aussage zu Fertilität und systemischer Melanomtherapie trifft zu?
  - Robuste klinische Daten zeigen, dass ICI‐Therapien die Fertilität dauerhaft stark beeinträchtigen.
  - BRAF‐ und MEK‐Inhibitoren sind sicher für die Fertilität und haben keine embryo‐ oder fetotoxischen Effekte.
  - Männer sind von Fertilitätsproblemen unter zielgerichteter Therapie grundsätzlich nicht betroffen.
  - Endokrine irAEs können indirekt die Fertilität beeinträchtigen und erfordern bei Kinderwunsch endokrinologische Mitbetreuung.
  - Eine Fertilitätsberatung vor Beginn der Therapie ist nur bei Frauen erforderlich.
5. Welche Empfehlung zum Fertilitätserhalt vor systemischer Melanomtherapie ist zutreffend?
  - Kryokonservierung von Ovarialgewebe ist bei Männern die Methode der Wahl.
  - Eine standardisierte Fertilitätsberatung sollte bei allen Patienten im reproduktiven Alter vor Therapiebeginn erfolgen.
  - GnRH‐Agonisten schützen nur Männer vor Chemotherapie‐induzierten Fertilitätsschäden.
  - Fertilitätsmaßnahmen sind nur bei metastasiertem Melanom indiziert.
  - Eine Kryokonservierung ist bei Frauen während laufender systemischer Therapie kontraindiziert und nie empfohlen.
6. Welche Aussage zur Messung der gesundheitsbezogenen Lebensqualität (HRQoL) bei Melanom‐Überlebenden ist korrekt?
  - HRQoL‐Instrumente erfassen ausschließlich körperliche Symptome und werden daher nur in der Akutphase eingesetzt.
  - Generische Instrumente sind auf Melanompatienten zugeschnitten und ersetzen onkologische HRQoL‐Fragebögen.
  - Eine Kombination aus generischen und onkologischen HRQoL‐Instrumenten ermöglicht sowohl Vergleichbarkeit mit der Allgemeinbevölkerung als auch die Erfassung krankheitsspezifischer Belastungen.
  - *Patient‐Reported Outcome*‐Instrumente (PROMs) sollten ausschließlich papierbasiert erhoben werden, um technische Barrieren zu vermeiden.
  - Subskalenanalysen sind nicht notwendig, da globale QoL‐Werte alle Dimensionen abbilden.
7. Welche Aussage zum Langzeitverlauf der Lebensqualität bei Melanom‐Überlebenden trifft am besten zu?
  - Nach erfolgreicher Primärtherapie erreichen nach einiger Zeit alle Patienten wieder die QoL‐Werte der Allgemeinbevölkerung.
  - Einschränkungen in Rollenfunktion, sozialer Teilhabe oder Vitalität können langfristig bestehen, selbst wenn die körperliche Autonomie erhalten bleibt.
  - Psychosoziale Belastungen wie Angst vor Rezidiv oder Veränderungen im Körperbild treten nur während der Therapie auf.
  - Geschlecht oder Alter bei Diagnose haben keinen Einfluss auf die QoL.
  - Globale QoL‐Scores erfassen zuverlässig alle funktionellen und psychosozialen Einschränkungen.
8. Welche Aussage zur Nachsorge und Therapieplanung nach kompletter Remission (CR) unter Immuntherapie beim metastasierten Melanom ist zutreffend?
  - Ein Progress nach frühem Absetzen der ICI‐Therapie bedeutet zwingend eine schlechte Prognose.
  - Das Absetzen der ICI nach CR ist in der klinischen Praxis äußerst selten.
  - Nachsorgeintervalle sind in allen Fällen risikoadaptiert und standardisiert.
  - Patienten mit PR (partieller Remission) zum Zeitpunkt des Therapieabbruchs haben ein höheres Progressionsrisiko als Patienten mit CR.
  - Eine Wiederaufnahme der ICI‐Therapie nach Progress ist nicht möglich.
9. Welche Komponenten sind typische Bestandteile von *Survivorship‐Care*‐Programmen für Melanompatienten?
  - Nur onkologische Nachsorge der körperlichen Funktionen
  - Survivorship‐Care beschränkt sich auf Patienten im Stadium I
  - Ausschließlich digitale Gesundheitsanwendungen
  - Nur medizinische Diagnostik ohne psychosoziale Aspekte
  - Psychosoziale Nachsorge, Überwachung auf Rezidive, Ressourcenkoordination sowie Information/*Empowerment*.
10. Welches der folgenden Statements zur onkologischen Rehabilitation von Melanompatienten in Deutschland ist richtig?
  - Alle Patienten haben grundsätzlich einen gesetzlichen Anspruch auf medizinische Rehabilitation, um soziale und berufliche Teilhabe wiederherzustellen oder zu sichern.
  - Eine Reha kann nur während der kurativen Therapie beantragt werden.
  - Medizinische Rehabilitation ist nur Patienten mit metastasiertem Melanom vorbehalten.
  - Die Wahl der Rehabilitationseinrichtung ist ausschließlich durch den behandelnden Arzt festgelegt.
  - Onkologische Rehabilitation zeigt bei Melanompatienten keinen nachgewiesenen Nutzen für Fatigue, körperliche Leistungsfähigkeit oder Lebensqualität.

Liebe Leserinnen und Leser, der Einsendeschluss an die DDA für diese Ausgabe ist der 30. November 2026.

Die richtige Lösung zum Thema Palmoplantare Pustulose: Entstehung, Differentialdiagnose und Therapie in Heft 4/2026 ist: 1c, 2b, 3d, 4c, 5a, 6b, 7b, 8b, 9b, 10c

Bitte verwenden Sie für Ihre Einsendung das aktuelle Formblatt auf der folgenden Seite oder aber geben Sie Ihre Lösung online unter http://jddg.akademie-dda.de ein.
